# Owner Observed Sleep Disturbances in Cavalier King Charles Spaniels with and Without Clinical Signs Compatible with a Chiari-like Malformation †

**DOI:** 10.3390/ani16142184

**Published:** 2026-07-14

**Authors:** Amanda J. Valentino, Natasha J. Olby

**Affiliations:** 1 Department of Clinical Sciences, College of Veterinary Medicine, North Carolina State University, Raleigh, NC 27607, USA; ajvalen2@ncsu.edu; 2 Comparative Medicine Institute, North Carolina State University, Raleigh, NC 27606, USA

**Keywords:** syringomyelia, sleep quality, sleep interruptions, sleep disruption

## Abstract

Chiari-like malformation (CM) and syringomyelia (SM) are prevalent in Cavalier King Charles Spaniels (CKCS). We hypothesized that CKCS with clinical signs compatible with CM+/-SM exhibit sleep disturbances. Thirty-seven CKCS with and without clinical signs compatible with CM+/-SM were included. No magnetic resonance imaging was required for inclusion. A questionnaire-based study was conducted using two validated instruments: CHASE to assess CM+/-SM severity and SNoRE 3.0 to evaluate sleep disturbances. Dogs with CHASE scores ≥7 were classified as having clinical signs. Nineteen dogs with clinical signs (median: 5 years, range: 3–10 years) and 18 without clinical signs (mean: 4.1 years, SD: 2.6) were included, with median CHASE scores of 13 (range: 7–18) and 1 (range: 0–6), respectively. Dogs with clinical signs had higher median SNoRE scores (median: 10, range: 6–30) than dogs without clinical signs (median: 7, range: 6–15). Sleep quality (*p*_adj_ = 0.008) and sleep interruptions due to dreaming (*p*_adj_ = 0.003) were significantly different between groups, with clinically affected dogs showing higher scores and greater variability. CKCS with clinical signs compatible with CM+/-SM show evidence of sleep disturbances, warranting confirmation with polysomnography and consideration in therapeutic management.

## Simple Summary

Chiari-like malformation (CM) is a skull abnormality common in small-breed dogs, resulting in overcrowding of the brain. This malformation is practically ubiquitous among the Cavalier King Charles Spaniel (CKCS) breed. This can cause disruption in cerebrospinal fluid flow, leading to the development of fluid-filled cysts in the spinal cord called syringomyelia (SM). Dogs with CM+/-SM can experience pain, scratching, and neurological problems, like people with a similar malformation. In people, this can result in sleep disturbances, but little is known about sleep in affected dogs. In this study, we used owner-completed questionnaires to compare sleep quality in CKCS with and without clinical signs compatible with CM+/-SM. Dogs with clinical signs had more disrupted sleep, including poor sleep quality and more frequent sleep interruptions. These findings suggest that sleep disturbances are a component of CM+/-SM in dogs and should be considered when managing their condition.

## 1. Introduction

Chiari-like malformation (CM) is a developmental disorder characterized by neuroparenchymal skeletal disproportion of the skull and craniocervical junctions, resulting in overcrowding of the foramen magnum and altered cerebrospinal fluid dynamics. It is common in small-breed dogs, although most often seen in the Cavalier King Charles Spaniel (CKCS) [1]. This condition can lead to the development of syringomyelia (SM) due to disruptions in cerebrospinal fluid (CSF) flow dynamics [2]. Clinical signs associated with CM+/-SM commonly include phantom scratching towards the head, neck, or shoulder, facial rubbing, scoliosis, ataxia, vocalization, and head and spinal pain [3,4].

In people, Chiari malformation type 1 (CM1) is a neuroanatomic disorder characterized by the herniation of the cerebellar tonsils through the foramen magnum, often associated with a reduced posterior fossa volume, which can result in the development of syringomyelia [5,6,7,8,9,10,11,12,13,14,15,16]. CM1 has been described in association with developmental variations of the skull, which may contribute to alterations in the morphology of the posterior fossa, and, notably, the canine ortholog may share features with these forms of Chiari malformation [12,17,18]. This disorder results in neural changes due to compression and crowding of the cerebellum, brainstem, and/or the upper cervical spinal cord at the craniocervical junction [5,14,16,18,19,20].

Symptoms of CM1 include headaches, pain, weakness, numbness, ataxia, cranial nerve dysfunction, and sleep disturbances [5,11,19,21,22]. Patients have been reported to experience increased sleepiness, insomnia, longer sleep latencies, shorter total sleep times, a greater incidence of restless legs syndrome, and a higher risk of sleep-disordered breathing [15]. Notably, sleep apnea has been acknowledged as a common clinical manifestation of CM1 [12,14,23].

Dogs can suffer from sleep disorders such as narcolepsy, rapid eye movement (REM) sleep behavior disorder, and sleep-disordered breathing [24,25]. In addition, chronic disorders such as osteoarthritis and canine cognitive dysfunction syndrome have been associated with sleep disruption [24,26,27]. Sleep disorders in dogs can manifest as interrupted sleep, sleeping with the chin elevated or mouth open, snoring, and choking or apneic episodes during sleep [28]. Disruption of sleep can lead to a range of negative outcomes, including cardiovascular and metabolic changes, as well as compromised physical and neurocognitive performance [12,29,30]. Sleep disturbances have been well documented in people with CM1, although prior studies have not systematically evaluated whether CKCS with clinical signs compatible with CM+/-SM exhibit similar sleep-related issues. It has been postulated that dogs with CM have sleep disturbances, although these are poorly defined. We aimed to investigate sleep quality in dogs with and without clinical signs compatible with the malformation using a validated, owner-completed questionnaire. We hypothesized that dogs with clinical signs compatible with CM+/-SM would exhibit more sleep disturbances than those without clinical signs, allowing for a more in-depth evaluation of sleep-related signs.

## 2. Materials and Methods

A survey was created using Qualtrics (Qualtrics LLC, Provo, UT, USA) to investigate sleep disturbances among client-owned dogs (Supplementary File S1). Participation was voluntary, and all owners completed an informed consent form to participate. Institutional Review Board approval was not sought for this study as questions were asked about dogs rather than their owners, classifying this work as “Not Human Subject Research”. The survey was distributed to owners of dogs that had previously participated in a study at North Carolina State University and to those whose owners had expressed interest in participating in past studies. The survey was also posted in two social media groups and emailed to Central Carolina CKCS Club members. Sample size was determined by case availability and owner participation during the study period.

Owners were asked to provide general information about their dog’s age, sex, other medical conditions, and medications, with a specific question regarding skin disease. Additionally, owners were asked whether their dog had a previous diagnosis or suspicion of CM+/-SM.

Data were obtained using two validated questionnaires. The Chiari-like Malformation and Syringomyelia Evaluation (CHASE) consists of 5 questions on a scale of 0–6, asking the owners to rate the severity of the dog’s signs related to CM+/-SM over the past 7 days in the categories of scratching, anxious, sensitive, uncomfortable, and restless. This generated an ordinal score that could be analyzed as continuous data but also allowed dogs to be categorized as either having clinical signs compatible with CM+/-SM or not, using the CHASE questionnaire threshold of 6 and under as not having clinical signs and 7 and above as showing signs. These thresholds were determined based on a previous evaluation of the CHASE questionnaire in a population of healthy, normal dogs that were not CKCS, as well as clinically affected CKCS [31]. Only a subset of dogs had undergone an MRI performed for an established diagnosis or screening for breeding. Chiari-like malformation with and without syringomyelia is nearly ubiquitous within the CKCS, including in clinically unaffected individuals [32,33]. Classification of clinical signs was solely made based on clinical scoring from the CHASE questionnaire because of the mismatch between clinical signs and findings on imaging that have been consistently reported in dogs with CM+/-SM [4,34,35,36].

The Sleep and Nighttime Restlessness Evaluation (SNoRE) 3.0 is a validated questionnaire tool for assessing sleep quality in dogs [27]. It consists of 6 questions divided into two categories: sleep quality and sleep interruptions caused by dreaming. Four questions were asked regarding sleep quality, including whether the dog falls asleep easily at bedtime, how often it gets up or paces, how frequently it needs to eliminate during the night, and how often it pauses breathing. Two questions addressed sleep interruptions caused by dreaming, asking how often vocalizations or twitching wake the dog. The owners rated all questions on a scale of 1 to 10.

Owners were also asked to report how much their dog snored at night over the previous seven days on a scale of 1–10 and whether their dog tended to sleep with its head propped up/elevated. Two open-ended questions were included for owners to describe any other abnormalities they wanted to report regarding their dogs’ sleeping patterns. Additionally, if the dog had started treatment for CM+/-SM, owners were asked whether the medications caused any changes in sleeping habits.

Dogs were excluded if they did not sleep in their owner’s room at night or had any skin conditions noted by the owner because of the potential impact on CHASE responses. Dogs included had to be CKCS, and only one dog per owner was included. Medical conditions reported by owners were evaluated individually for their potential to disrupt sleep or alter the CHASE score. All data used in this analysis is available in Supplementary File S2.

Statistical analysis was performed using JMP Pro 18 (SAS Institute, Cary, NC, USA). Dogs were grouped into two categories: those with clinical signs compatible with CM+/-SM (CHASE score of 7 or greater) and those without (CHASE score < 7). Summary data on age, sex, CHASE, and SNoRE 3.0 scores were prepared for each group. A Shapiro–Wilk test was used to determine the normality of continuous data; normal data were expressed as the mean and standard deviation, non-normal data were expressed as the median and range, categorical data were expressed as proportions. Age, sex and pain medication (yes or no) were compared between the two groups using a Wilcoxon rank-sum test (age) or contingency tables (sex and pain medications) with a Fisher’s Exact test. Given differences in treatment of CM+/-SM signs between these two groups, we examined whether pain medications alone altered sleep by grouping dogs according to pain medication (yes or no) and comparing the total SNoRE score between groups with a Wilcoxon rank-sum test.

The relationship between clinical signs compatible with CM+/-SM and sleep was examined through analyses of CHASE and SNoRE scores. With dogs grouped as clinically affected or not, the SNoRE total score and scores for the two SNoRE categories (sleep quality and sleep interruptions caused by dreaming) were compared between groups using a Wilcoxon rank-sum test. To explore the relationship between the severity of signs and the severity of sleep disturbance further, the relationship between CHASE and SNoRE 3.0 scores was evaluated across the entire study cohort using linear regression. Finally, responses to individual questions were plotted graphically for visual evaluation to determine whether any difference detected was driven by a particular question. The age of CKCS with and without twitching at night was compared using a Wilcoxon Rank Sum test to investigate whether the twitching was caused by age-associated myoclonus. The responses to head position during sleep and snoring were compared by the construction of contingency tables and chi-square analysis. A Holm-Bonferroni correction was applied to account for multiple comparisons, and adjusted *p* values were reported as *p*_adj_.

## 3. Results

Fifty-four owners completed the questionnaire. Fourteen dogs were excluded because they did not sleep in the owner’s room at night, and one owner completed the questionnaire for two dogs, so the second dog was removed. One dog was excluded because it was a different breed, and another was excluded due to food allergies that caused skin irritation. One dog did not have a reported response on whether it slept with its head elevated, leaving 36 dogs with complete surveys and one dog lacking a single response.

Eighteen dogs fit our criteria for not having clinical signs compatible with CM+/-SM. These dogs had a mean age of 4.1 years (SD: 2.6) and a median CHASE score of 1 (range: 0–6). Eight were neutered males, eight were spayed females, and two were intact females. This group included one dog with suspected CM+/-SM, three dogs diagnosed with CM and SM on magnetic resonance imaging (MRI), and 14 dogs that were considered normal by their owners and had not undergone advanced imaging. Comorbidities reported included myxomatous mitral valve disease (2), protein-losing enteropathy/deafness/heart murmur (1), keratoconjunctivitis sicca (1), and exocrine pancreatic insufficiency/urinary incontinence (1). The dog with urinary incontinence did not have an increase in elimination overnight. Medications and supplements included topical eye medications, supplements, pregabalin, prednisone, pimobendan, Atopica, enalapril, spironolactone, Incurin, pancreatic enzymes, Crananidin, Coballequin, gabapentin, omeprazole, Fortiflora, Myos supplement, Dasuquin, and Antinol.

Nineteen dogs met the CHASE threshold to be categorized as having clinical signs compatible with CM+/-SM. These dogs had a median age of 5 years (range: 3–10 years) with a mean CHASE score of 12.3 (SD: 3.7). Seven dogs were neutered males, one was an intact male, and 11 were spayed females. This group included 13 dogs diagnosed with CM on MRI and six that had not undergone advanced imaging. Of the 13 dogs diagnosed with CM on MRI, 10 also had SM. Comorbidities in this group of dogs included primary secretory otitis media (2), macrothrombocytopenia (1), myxomatous mitral valve disease (1), arthritis (1), loose and soft stool (1), flycatcher’s syndrome (1), dry eye (1), and being deaf with a heart murmur (1). The medications and supplements the dogs were taking at the time included trazodone, fluoxetine, pregabalin, tramadol, cyclosporine eye drops, tacrolimus eye drops, pimobendan, Rimadyl/Carprofen, probiotics, gabapentin, and CBD oil.

No significant differences in age (*p* = 0.15) or sex (*p* = 0.44) were observed between groups.

Twenty-four dogs were not on pain medication at the time of this survey, and 13 were taking one or more of the following medications: pregabalin, gabapentin, carprofen, and/or tramadol. Medications controlled signs effectively in two of these dogs, and they had CHASE scores of <7 at the time of the survey and were therefore categorized as not clinically affected. Eight owners reported a change in their dogs’ sleep patterns when they started on medication, noting that their dogs slept better, they were calmer and more comfortable, did not wake up to scratch, slept more restfully, and required fewer adjustments to their position during the night, resulting in deeper sleep. Among these dogs, one owner reported that their dog woke up more often and abruptly since starting the medication, while another reported that their dog slept more during the day, and another specifically noted that their dog woke up earlier and scratched persistently until the owners got up.

**Figure 1:**
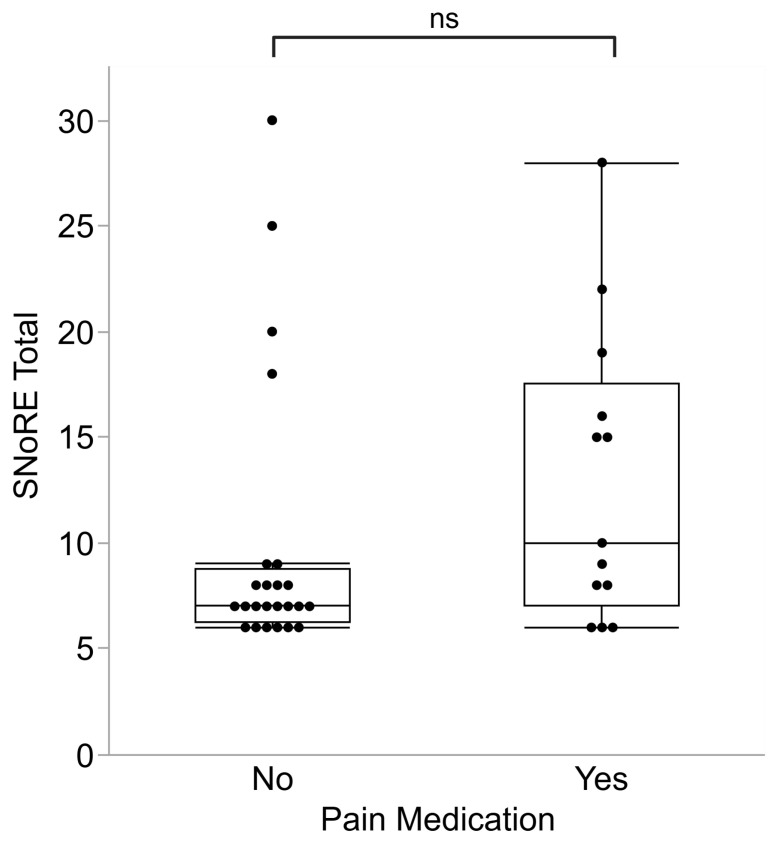
Box plot of SNoRE Total scores in dogs receiving or not receiving pain medication at the time of questionnaire completion. Dots represent individual dogs. The horizontal line indicates the median. ns = not significant.

Of the thirteen dogs that were receiving pain medication at the time of this survey: eight dogs were on pregabalin, four were on gabapentin, two were on carprofen, and two were on tramadol (one being only given as needed). Two of these dogs were receiving multiple pain medications at the same time: one receiving pregabalin and tramadol and the other receiving carprofen and gabapentin. A larger proportion of dogs with clinical signs compatible with CM+/-SM were on pain medications (11 out of 19) compared to those without clinical signs (2 out of 18) (*p* = 0.005). Given this treatment difference between groups, we compared SNoRE scores between dogs based on treatment with pain medications and found no significant difference between groups (*p* = 0.11). (Figure 1) A similar result was observed when dogs were compared based on treatment with gabapentinoids alone (*p* = 0.15).

**Figure 2:**
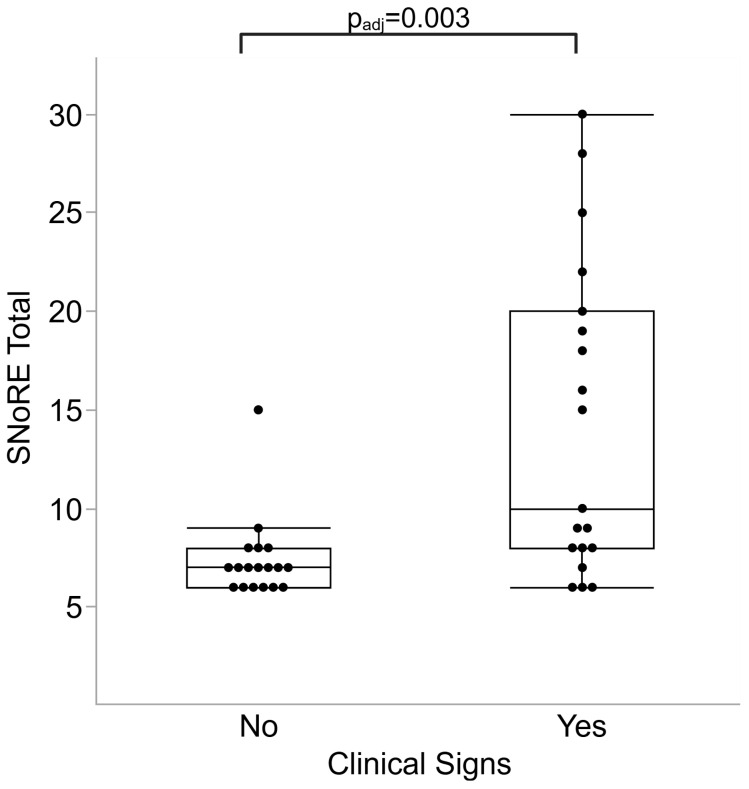
Box plot of SNoRE Total scores in dogs with and without clinical signs compatible with a Chiari-like malformation +/− syringomyelia. Dots represent individual dogs. The horizontal line indicates the median. The adjusted *p*-value for the group comparison is shown above the bracket.

When evaluating the total SNoRE score, dogs with clinical signs compatible with CM+/-SM had a median score of 10 (range: 6–30), whereas those without clinical signs had a median score of 7 (range: 6–15). This difference was significant (*p* ≤ 0.001, *p*_adj_ = 0.003) (Figure 2) The group without clinical signs had one outlier with a total SNoRE score of 15, the only score exceeding 10. The owner of this dog reported that it was suspected of having clinical signs compatible with CM+/-SM by the veterinarian. This dog had no other listed medical conditions and was receiving gabapentin and omeprazole at the time of this survey. The SNoRE scores were separated into two clusters in the group of dogs with clinical signs compatible with CM+/-SM. Nine dogs in the group with clinical signs had scores above 10, while the remaining 10 dogs had scores that were comparable to the group that did not have clinical signs (Figure 2).

**Figure 3:**
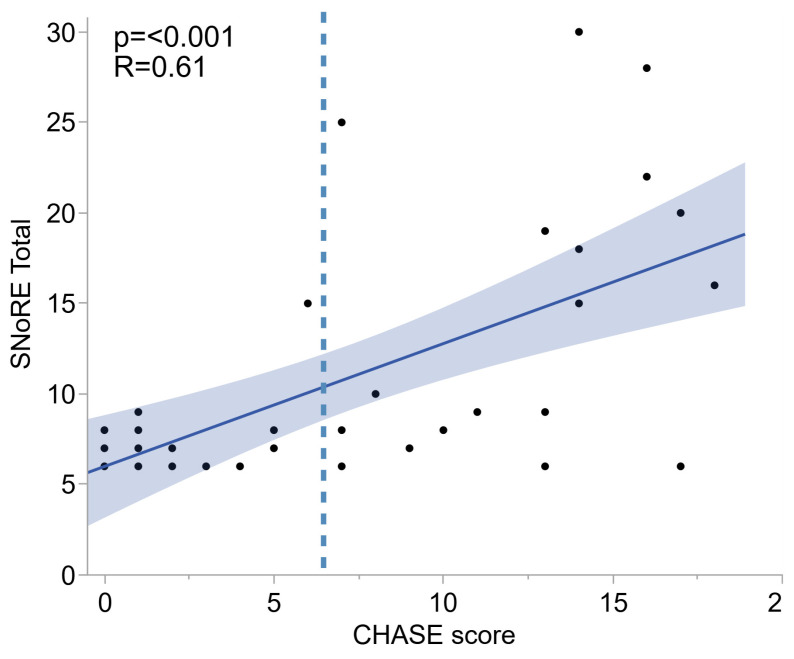
Scatter plot of SNoRE Total score versus CHASE score. A line of best fit (solid dark blue) is shown with the 95% confidence interval (blue shading). The vertical dashed blue line indicates the division between dogs with and without clinical signs compatible with a Chiari-like malformation +/− syringomyelia. The corresponding *p*-value and R-value are displayed in the upper left corner.

After determining that a significant difference exists between dogs with and without clinical signs compatible with the malformation, we assessed whether these scores were directly correlated. A moderately strong positive correlation (*p* ≤ 0.001, R = 0.61), was seen between the CHASE score and the total SNoRE score (Figure 3).

**Figure 4:**
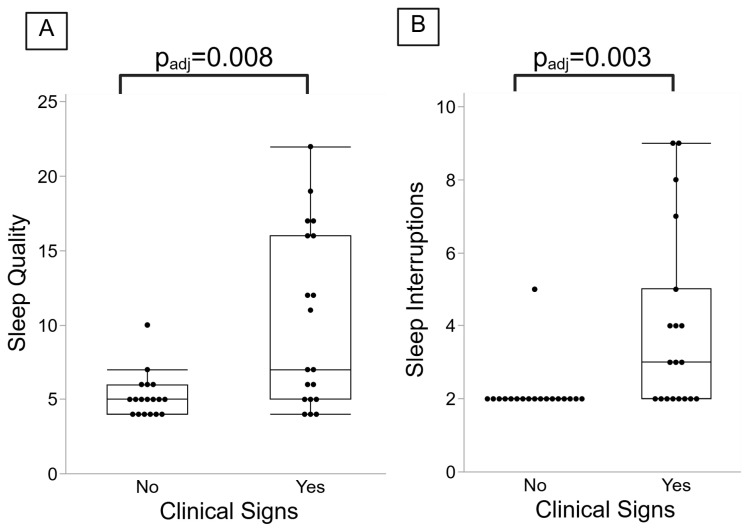
Box plots of the SNoRE questionnaire categories: sleep quality (**A**) and sleep interruptions due to dreaming (**B**), in dogs with and without clinical signs compatible with a Chiari-like malformation +/− syringomyelia. Dots represent individual dogs; horizontal lines indicate medians. Corresponding adjusted *p*-values are shown above the brackets.

When broken down into their respective categories of sleep quality and sleep interruptions caused by dreaming, the significant difference in total SNoRE scores was driven by responses in both areas, with significant differences between groups for quality (*p*_adj_ = 0.008), and interruptions (*p*_adj_ = 0.003) (Figure 4). The outlier noted in the total SNoRE score was also an outlier in both categories, as was the spread of SNoRE scores in clinically affected dogs, with a subset having severe problems and others scoring more normally.

**Figure 5:**
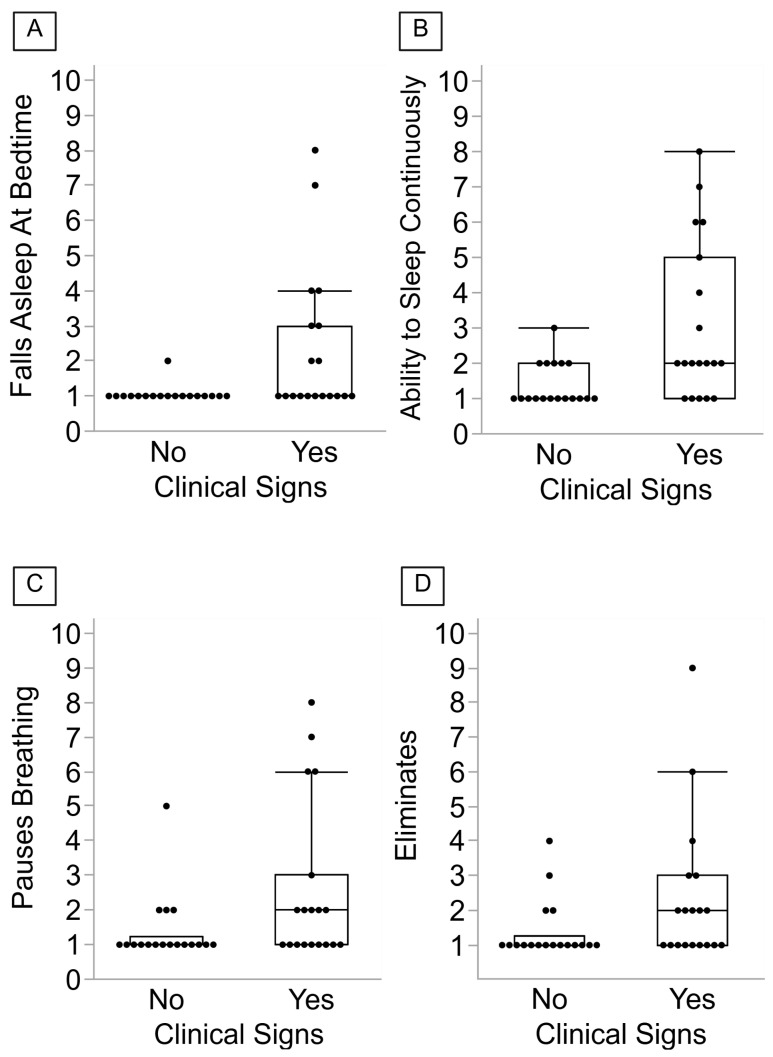
Box plots of SNoRE questions within the sleep quality category: ability to fall asleep (**A**), ability to sleep continuously (**B**), pauses in breathing (**C**), and need to eliminate overnight (**D**). Dots represent individual dogs; horizontal lines indicate the medians.

**Figure 6:**
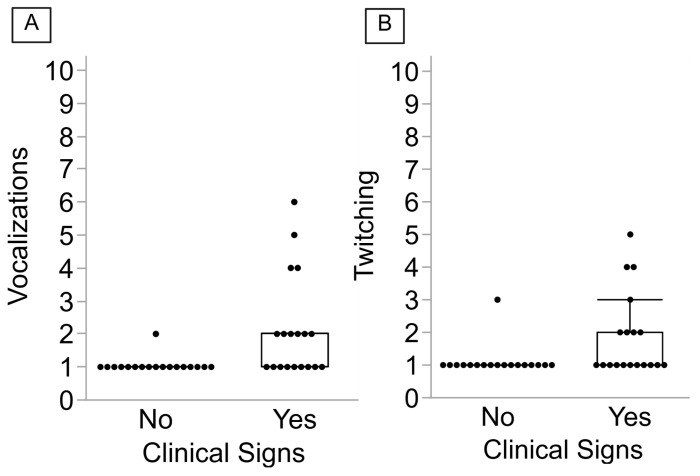
Box plots of SNoRE questions within the sleep interruptions caused by dreaming category: vocalizations that wake the dog up (**A**) and twitching that wakes the dog up (**B**). Dots represent individual dogs; horizontal lines indicate the medians.

The responses to individual questions are shown in Figure 5 (sleep quality questions) and Figure 6 (sleep interruptions caused by dreaming questions) with data provided in Table 1 and Table 2. These data demonstrate that group differences are not being driven by one question or type of sleep disturbance. Notably, age-associated myoclonus has commonly been reported among older CKCS regardless of CM+/-SM status [37]. The median twitching score among all dogs was 1, with 9 dogs exhibiting a score greater than 1. Dogs with a twitching score of 1 had a median age of 4 years (range: 1–10 years), while those with a score greater than 1 also had a median age of 4 years (range: 3–6 years). There was no significant difference in age between dogs with higher versus lower twitching scores (*p* = 0.68), suggesting that the observed twitching behavior during sleep is unlikely to be related to age-associated myoclonus. This is further supported by the age of the CKCS in this study (mean: 4.1 years, SD: 2.6).

**Figure 7:**
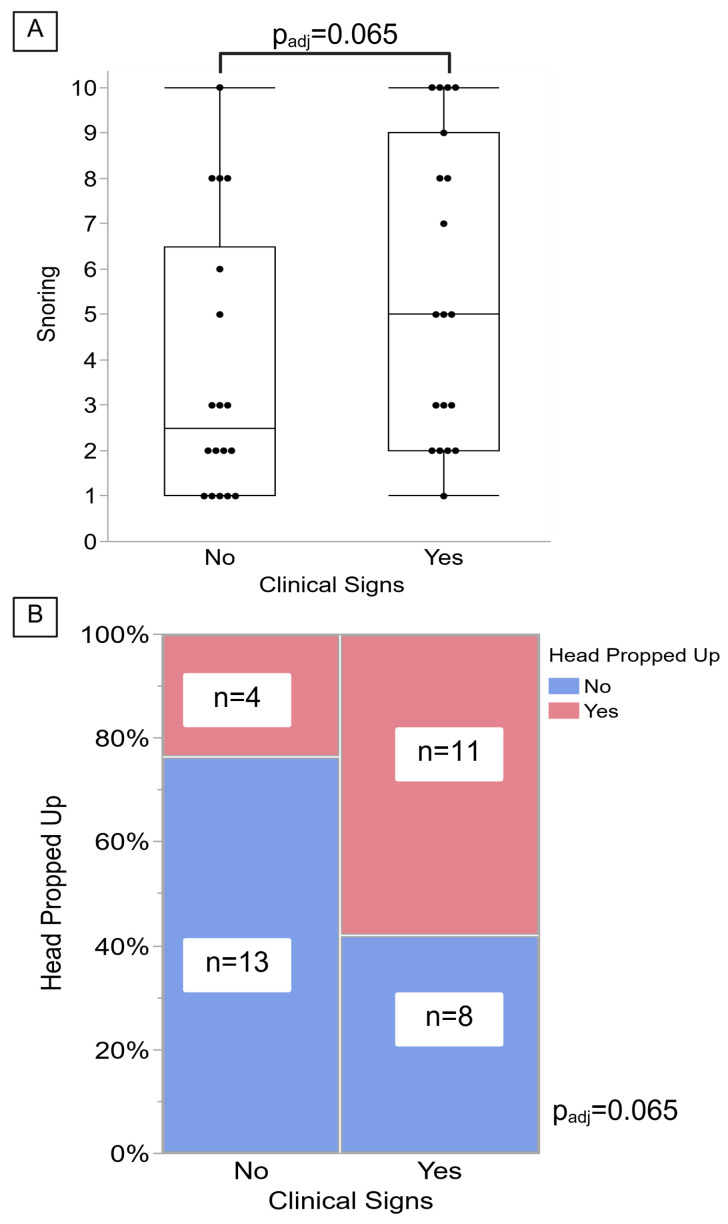
Box plot (**A**) showing the prevalence of snoring in dogs with and without clinical signs compatible with a Chiari-like malformation +/− syringomyelia. Each dot represents an individual dog; horizontal lines indicate medians. Corresponding adjusted *p*-value is shown above the brackets. A contingency table (**B**) depicting whether dogs sleep with their heads propped up by clinical status. The adjusted *p*-value is displayed in the bottom right corner. A color key is provided in the upper right corner, and each section is labeled with the number of dogs (n).

Dogs that had clinical signs compatible with CM+/-SM had a higher median snoring score (median: 5, range: 1–10) compared to those without clinical signs (median: 2.5, range: 1–10). This resulted in a significant difference between the two groups, but once corrected for multiple comparisons, it did not reach this threshold (*p* = 0.033, *p*_adj_ = 0.065). Similarly, 57.9% of dogs with clinical signs tended to sleep with their heads propped up, compared to only 23.5% of dogs without clinical signs. This behavior was also significantly associated with the presence of clinical signs compatible with CM+/-SM, until corrected for multiple comparisons (*p* = 0.034, *p*_adj_ = 0.065). Of the four dogs without clinical signs reported to sleep with their heads up, one was suspected to have CM+/-SM, and was the outlier noted in each of the categories in the SNoRE 3.0. (Figure 7).

## 4. Discussion

Chiari-like malformation in dogs shares similarities with CM1 in people, leading us to investigate whether sleep disturbances exist among dogs with this condition. Based on the SNoRE 3.0 questionnaire, clinically affected dogs experienced more sleep disturbances, clustering into two distinct groups: those with signs of sleep disruption and those without. The difference in SNoRE scores was driven by differences across both categories of the questionnaire and individual questions, suggesting that sleep disturbances manifest in multiple ways.

In people and dogs with this condition, sleep disturbances may stem from compression of the brainstem, cervical spinal cord and the more caudal cranial (IX-XII) nerves, resulting in respiratory compromise, cranial nerve dysfunction, and disruptions in sleep regulation [8,9,12,14,38,39,40]. Studies in people with CM1 have reported insomnia, with one study reporting sleep latency nearly three times longer than in controls [15]. Similarly, owners in our study reported that dogs with clinical signs compatible with CM+/-SM took longer to fall asleep. People with CM1 also experience shorter habitual sleep duration and a higher prevalence of restless legs syndrome [15]. In our population, more dogs with clinical signs woke and paced during the night, which might reflect difficulty finding a comfortable position due to chronic pain. Sleep disturbances, including insomnia and frequent waking, are commonly associated with pain in both humans and animals, as supported by questionnaire data and activity monitoring [26,41,42].

Owners also noted increased nighttime elimination in dogs with clinical signs compatible with CM+/-SM. While this might be secondary to more frequent awakenings, increased daytime and nighttime frequency of urination are documented in people with SM, a frequent comorbidity in CM [43]. However, we could not rule out other non-neurological causes of increased urinary frequency in our study population, because while owners were asked to list other medical conditions, full medical records were not obtained for these dogs.

Dogs that had clinical signs compatible with CM+/-SM were more likely to exhibit breathing pauses during sleep. Sleep-disordered breathing has been reported with a prevalence of approximately 50–70% in individuals with CM1, with frequency depending on assessment criteria [8,10,14,15,18,21,39]. Sleep-disordered breathing patterns reported include obstructive, central, mixed, and hypopneas [8,10,12,13,19,38,44,45,46]. Compression of the brainstem can alter ventilatory control through impairment of central chemoreception, which may cause decreased responsiveness to carbon dioxide and disruption of feedback mechanisms [8,18,44,47,48]. This might also result in brief arousals due to hypoventilation, elevated end-tidal carbon dioxide levels, and reduced respiratory rates, resulting in sleep fragmentation [13,47]. Another possibility is dysfunction of the pontomedullary respiratory network, particularly the ventral medullary group that is responsible for respiratory rhythm generation [44]. Additionally, cervical SM can cause compression of the phrenic motor neurons and reduced blood supply to the brainstem, both of which might contribute to this dysfunction [12,49].

Obstructive sleep apnea is associated with brachycephalic breeds such as Pugs, French Bulldogs, English Bulldogs, and CKCS, and is characterized by snoring, recurrent partial and/or complete obstruction of the upper airway during sleep, intermittent oxyhemoglobin desaturations, and sleep disruption [25,50,51]. In CKCS, snoring might result from craniofacial structural abnormalities. In a morphometric study in CKCS with pain from CM+/-SM or that had a large syrinx, osseous insufficiency resulting in rostral flattening, and a closer proximity of the soft palate to the cranial base were noted, which might further compromise airway integrity and contribute to snoring [50]. Obstructive sleep apnea has also been reported among people with CM1, possibly due to compression of the cranial nerves IX, X, and XI, resulting in decreased tone of the pharyngeal, laryngeal, and dilating muscles or loss of upper airway sensation, allowing for negative thoracic pressure that occurs with inhalation to cause upper airway collapse [8,12,18,23,46].

During REM sleep, dogs can exhibit limb paddling, twitching, yelping, and muffled barking, and this can occur without disrupting their sleep. However, in our study, dogs with clinical signs compatible with CM+/-SM experienced vocalization and twitching severe enough to disrupt their sleep. In people with CM1, it is proposed that REM sleep behavior disorder occurs because of brainstem compression leading to dysfunction of the reticular activating system and neuron groups responsible for inhibitory neuronal control during REM sleep, which can result in hyperarousal, increased wakefulness, and sleep fragmentation [10,44]. Similar brainstem dysfunction in CM might underlie this increase in activity that disrupts sleep among affected dogs.

Some dogs with CM+/-SM prefer sleeping with their heads elevated, a behavior that is more common in those with a large syrinx [52]. Elevation of the head to 30 degrees is recommended when managing dogs with increased intracranial pressure (ICP) based on human head injury guidelines. It has been demonstrated in people that lying flat increases ICP, while elevating the head to 30 degrees lowers ICP, through enhanced venous return while maintaining cerebral perfusion [53,54]. We hypothesize that dogs with CM+/-SM seek head elevation when sleeping to avoid increases in ICP and the accompanying headaches.

Sleep-disordered breathing has been described as an initial manifestation of CM1 in people, even without other neurologic signs [14,21,55]. In our study, one dog was an outlier in both categories of the SNoRE 3.0 in the group of dogs without clinical signs. This dog was suspected to have CM+/-SM and was being successfully treated with gabapentin and omeprazole at the time of this survey with CHASE scores below the threshold used to separate clinically affected and normal dogs. This dog might reflect a presentation that was characterized primarily by sleep disturbances, similar to that seen in people, although other factors could also explain the sleep disturbances.

Impaired sleep is associated with a wide range of adverse physical, cognitive, and psychological effects [15]. These include increases in cardiovascular diseases, oxidative stress, inflammation, endothelial dysfunction, metabolic changes, and carotid body activity, as well as decreased nitric oxide bioavailability, leading to negative effects on the brain, cerebrovasculature, and heart [14,31,56,57,58,59]. More specifically, sleep-related breathing disturbances might exacerbate neuropathology and clinical signs among dogs with CM+/-SM. Hypercapnia and increased respiratory effort against an obstructed airway can cause cerebral vasodilation, resulting in transient intracranial hypertension, potentially increasing cerebellar herniation, compressing neural tissue, and promoting the development or worsening of SM or hydrocephalus [5,13,45,46,60,61]. Among dogs with neuropathic pain syndromes, sleep disruption might be especially harmful, because sleep fragmentation can exacerbate pain, increase hypersensitivity, and alter neurotransmitter modulation, possibly contributing to the development or persistence of chronic pain [62,63]. These possible adverse effects emphasize the importance of evaluating sleep in the therapeutic plan for dogs with CM+/-SM and pain.

This study was intentionally designed to use owner-reported clinical signs for both CM+/-SM and sleep. The validated questionnaires used are designed to capture the signs that owners witness in the home environment. Capturing signs of pain is notoriously difficult, and it is well established that the MRI findings do not correlate well with clinical signs, as identified both by veterinarians in hands-on examinations and by owner reports in CM+/-SM [4,35,36,52,64]. The gold standard for sleep evaluation and quantification is polysomnography, but this is challenging and time-consuming to perform in dogs, and the SNoRE 3.0 was validated against both activity monitors and polysomnography [27].

Our study had several limitations. First, we relied on the owners for accurate reporting of their dog’s current state and medical history. This study did not include direct examinations of dogs, such as evaluations for brachycephalic obstructive airway syndrome, respiratory grading, obesity, environmental conditions, and other comorbidities, which could also influence sleep. Comorbidities that were present within this population were unable to be investigated in depth because of small sample sizes. In addition, we did not have brain MRI scans for every dog in the study and so we cannot confirm their imaging status, either positive or negative regarding CM and SM; rather we identified the population based on the presence or absence of typical signs of CM+/-SM. A subset of dogs in this study were receiving medications for pain or behavioral signs. Although there were no apparent effects of medications on sleep in our cohort, and overall data on their effects on sleep in dogs is limited, it is important to acknowledge that several of these agents have the potential to influence sleep. Owner bias and recall bias might have influenced the questionnaire responses. Owners who are hypervigilant or have a dog with more medical needs may be more aware of potential issues that might seem less concerning to other owners. Similarly, the frequency and severity of reported sleep disturbances may not fully reflect the dog’s objective sleep patterns if owners are not accurately remembering past events. Finally, hypersomnia and excessive daytime somnolence have been reported in those with CM1, but SNoRE 3.0 does not evaluate these aspects of sleep [8,60].

This article is a revised and expanded version of an abstract presented as an oral research presentation titled “Owner Observed Sleep Disturbances Between Cavalier King Charles Spaniels with and without Symptomatic Chiari-like Malformation” at the American College of Veterinary Internal Medicine Forum in Louisville, Kentucky in June of 2025 [65].

## 5. Conclusions

We conclude that CKCS with clinical signs presumed to be associated with CM+/-SM also exhibit sleep disturbances. Veterinarians should discuss the signs suggestive of sleep disturbances and sleep-disordered breathing, such as snoring, apneas, and daytime somnolence, with dog owners. Treatment options for sleep disorders are limited in dogs because interest in this medical problem has been limited historically. Future studies should focus on using objective diagnostic methods such as polysomnography to validate the presented findings.

## Figures and Tables

**Table 1:** Summary of median and range for the category of sleep quality questions in dogs with and without clinical signs compatible with a Chiari-like malformation +/− syringomyelia.

Question	Clinical Signs	Median	Range
Ability to sleep at bedtime	No	1	1–2
	Yes	1	1–8
Ability to sleep continuously	No	1	1–3
	Yes	2	1–8
Pauses breathing	No	1	1–5
	Yes	2	1–8
Need to eliminate during the night	No	1	1–4
	Yes	2	1–9

**Table 2:** Summary of median and range for the category of sleep interruptions caused by dreaming questions in dogs with and without clinical signs compatible with a Chiari-like malformation +/− syringomyelia.

Question	Clinical S1.	Median	Range
Vocalization that wakes the dog up	No	1	1–2
	Yes	2	1–6
Twitching that wakes the dog up	No	1	1–3
	Yes	1	1–5

## Data Availability

The original contributions presented in this study are included in the Supplementary Materials. Further inquiries can be directed to the corresponding author.

## Supplementary Materials

The following supporting information can be downloaded at: https://www.mdpi.com/article/10.3390/ani16142184/s1, Supplementary File S1: Sleep Study Questionnaire, Supplementary File S2: Data File.

## Abbreviations

The following abbreviations are used in this manuscript:

CHASE	Chiari-like Malformation and Syringomyelia Evaluation
CKCS	Cavalier King Charles Spaniels
CM	Chiari-like malformation
CM1	Chiari malformation type 1
ICP	Intracranial pressure
MRI	Magnetic resonance imaging
REM	Rapid eye movement
SM	Syringomyelia
SNoRE	Sleep and Nighttime Restlessness Evaluation

## References

[1] Cerda-Gonzalez S., Olby N.J., McCullough S., Pease A.P., Broadstone R., Osborne J.A. (2009). Morphology of the Caudal Fossa in Cavalier King Charles Spaniels. Vet. Radiol. Ultrasound.

[2] Cerda-Gonzalez S., Olby N.J., Broadstone R., McCullough S., Osborne J.A. (2009). Characteristics of Cerebrospinal Fluids Flow in Cavalier King Charles Spaniels Analyzed Using Phase Velocity Cine Magnetic Resonance Imaging. Vet. Radiol. Ultrasound.

[3] Cappello R., Rusbridge C., Chiari-Like Malformation and Syringomyelia Working Group (2007). Report from the Chiari-Like Malformation and Syringomyelia Working Group Round Table. Vet. Surg..

[4] Rusbridge C., McFadyen A.K., Knower S.P. (2019). Behavioral and clinical signs of Chiari-like malformation-associated pain and syringomyelia in Cavalier King Charles spaniels. J. Vet. Intern. Med..

[5] Voutsas G., St-Laurent A., Hutchinson C., Amin R., Drake J., Narang I. (2021). The efficacy of neurosurgical intervention on sleep-disordered breathing in pediatric patients with Chiari malformation type I. J. Neurosurg. Pediatr..

[6] do Vale J.M., Silva E., Pereira I.G., Marques C., Sanchez-Serrano A., Torres A.S. (2013). Chiari malformation and central sleep apnea syndrome: Efficacy of treatment with adaptive servo-ventilation. J. Bras. Pneumol..

[7] Tsara V., Serasli E., Kimiskidis V., Papagianopoulos S., Katsaridis V., Fylaktakis M., Christaki P., Kazis A. (2005). Acute respiratory failure and sleep-disordered breathing in Arnold–Chiari malformation. Clin. Neurol. Neurosurg..

[8] Dauvilliers Y., Stal V., Abril B., Coubes P., Bobin S., Touchon J., Escourrou P., Parker F., Bourgin P. (2007). Chiari malformation and sleep related breathing disorders. J. Neurol. Neurosurg. Psychiatry.

[9] Botelho R.V., Bittencourt L.R.A., Rotta J.M., Tufik S. (2005). Adult Chiari malformation and sleep apnoea. Neurosurg. Rev..

[10] Henriques-Filho P.S.A., Pratesi R. (2008). Sleep apnea and REM sleep behavior disorder in patients with Chiari malformations. Arq. Neuro-Psiquiatr..

[11] Losurdo A., Dittoni S., Testani E., Blasi C.D., Scarano E., Mariotti P., Paternoster G., Di Rocco C., Massimi L., Della Marca G. (2013). Sleep Disordered Breathing in Children and Adolescents with Chiari Malformation Type I. J. Clin. Sleep Med..

[12] Abel F., Tahir M.Z. (2019). Role of sleep study in children with Chiari malformation and sleep disordered breathing. Child’s Nerv. Syst..

[13] Becker H.F. (2005). Adult Chiari malformation and sleep apnoea. Neurosurg. Rev..

[14] Ferré Á., Poca M.A., de la Calzada M.D., Moncho D., Romero O., Sampol G., Sahuquillo J. (2017). Sleep-Related Breathing Disorders in Chiari Malformation Type 1: A Prospective Study of 90 Patients. Sleep.

[15] Watson N.F., Buchwald D., Noonan C., Goldberg J., Maravilla K., Ellenbogen R.G. (2010). Sleep in patients with Chiari-I malformations. Sleep Biol. Rhythms.

[16] Nishikawa M., Sakamoto H., Hakuba A., Nakanishi N., Inoue Y. (1999). Pathogenesis of Chiari Malformation: A Morphometric Study of the Posterior Cranial Fossa. Neuroradiol. J..

[17] Cross H.R., Cappello R., Rusbridge C. (2009). Comparison of cerebral cranium volumes between cavalier King Charles spaniels with Chiari-like malformation, small breed dogs and Labradors. J. Small Anim. Pract..

[18] Vagianou F., Khirani S., Denis T.D.S., Beccaria K., Amaddeo A., Breton S., James S., Paternoster G., Arnaud E., Zerah M. (2022). Impact of sleep-disordered breathing on the management of children with Chiari malformation type I. Pediatr. Pulmonol..

[19] Khatwa U., Ramgopal S., Mylavarapu A., Prabhu S.P., Smith E., Proctor M., Scott M., Pai V., Zarowski M., Kothare S.V. (2013). MRI Findings and Sleep Apnea in Children with Chiari I Malformation. Pediatr. Neurol..

[20] Urbizu A., Poca M., Vidal X., Rovira A., Sahuquillo J., Macaya A. (2014). MRI-based Morphometric Analysis of Posterior Cranial Fossa in the Diagnosis of Chiari Malformation Type I. J. Neuroimaging.

[21] Jarrell M., Caudill C., Haji F., Leon T., Rozzelle C.J., Maddox M.H., Rocque B.G. (2024). Sleep-disordered breathing in children with Chiari type I malformation. J. Neurosurg. Pediatr..

[22] Tubbs R.S., Beckman J., Naftel R.P., Chern J.J., Wellons J.C., Rozzelle C.J., Blount J.P., Oakes W.J. (2011). Institutional experience with 500 cases of surgically treated pediatric Chiari malformation Type I: Clinical article. J. Neurosurg. Pediatr..

[23] Kitamura T., Miyazaki S., Kadotani H., Kanemura T., Okawa M., Tanaka T., Komada I., Hatano T., Suzuki H. (2014). Type I Chiari malformation presenting central sleep apnea. Auris Nasus Larynx.

[24] Mondino A., Delucchi L., Moeser A., Cerdá-González S., Vanini G. (2021). Sleep Disorders in dogs: A Pathophysiological and Clinical Review. Top. Companion Anim. Med..

[25] Hendricks J.C., Kline L.R., Kovalski R.J., O’Brien J.A., Morrison A.R., Pack A.I. (1987). The English bulldog: A natural model of sleep-disordered breathing. J. Appl. Physiol..

[26] Gruen M.E., Samson D.R., Lascelles B.D.X. (2019). Functional linear modeling of activity data shows analgesic-mediated improved sleep in dogs with spontaneous osteoarthritis pain. Sci. Rep..

[27] Mondino A., Ludwig C., Menchaca C., Russell K., Simon K.E., Griffith E., Kis A., Lascelles B.D.X., Gruen M.E., Olby N.J. (2023). Development and validation of a sleep questionnaire, SNoRE 3.0, to evaluate sleep in companion dogs. Sci. Rep..

[28] Pohl S., Roedler F.S., Oechtering G.U. (2016). How does multilevel upper airway surgery influence the lives of dogs with severe brachycephaly? Results of a structured pre- and postoperative owner questionnaire. Vet. J..

[29] Beaudin A.E., Waltz X., Hanly P.J., Poulin M.J. (2017). Impact of obstructive sleep apnoea and intermittent hypoxia on cardiovascular and cerebrovascular regulation. Exp. Physiol..

[30] Trosman I., Trosman S.J. (2017). Cognitive and Behavioral Consequences of Sleep Disordered Breathing in Children. Med. Sci..

[31] Valentino A., Moore S.A., Fitzgerald S., Kennedy S., Farrell A., Costello M., Roynard P., Olby N.J. (2026). Development and validation of the Chiari-like malformation and syringomyelia evaluation: The CHASE questionnaire. J. Vet. Intern. Med..

[32] Couturier J., Rault D., Cauzinille L. (2008). Chiari-like malformation and syringomyelia in normal cavalier King Charles spaniels: A multiple diagnostic imaging approach. J. Small Anim. Pract..

[33] Harcourt-Brown T.R., Campbell J., Warren-Smith C., Jeffery N.D., Granger N.P. (2015). Prevalence of Chiari-like Malformations in Clinically Unaffected Dogs. J. Vet. Intern. Med..

[34] Cerda-Gonzalez S., Olby N.J., Griffith E.H. (2016). Longitudinal Study of the Relationship among Craniocervical Morphology, Clinical Progression, and Syringomyelia in a Cohort of Cavalier King Charles Spaniels. J. Vet. Intern. Med..

[35] Tirrito F., Cozzi F., Bonaldi M., Corazzo S., Contiero B., Lombardo R. (2022). Ventriculomegaly in Cavalier King Charles Spaniels with Chiari-like malformation: Relationship with clinical and imaging findings. J. Vet. Med. Sci..

[36] Sparks C.R., Cerda-Gonzalez S., Griffith E.H., Lascelles B.D.X., Olby N.J. (2018). Questionnaire-based Analysis of Owner-reported Scratching and Pain Signs in Cavalier King Charles Spaniels Screened for Chiari-like Malformation and Syringomyelia. J. Vet. Intern. Med..

[37] Rotter C., Whittaker D., Rusbridge C. (2022). Myoclonus in older Cavalier King Charles Spaniels. J. Vet. Intern. Med..

[38] Botelho R.V., Bittencourt L.R.A., Rotta J.M., Tufik S. (2010). The effects of posterior fossa decompressive surgery in adult patients with Chiari malformation and sleep apnea: Clinical article. J. Neurosurg..

[39] Botelho R.V., Bittencourt L.R.A., Rotta J.M., Tufik S. (2003). A prospective controlled study of sleep respiratory events in patients with craniovertebral junction malformation. J. Neurosurg..

[40] Nogués M., Gené R., Benarroch E., Leiguarda R., Calderón C., Encabo H. (1999). Respiratory disturbances during sleep in syringomyelia and syringobulbia. Neurology.

[41] Knazovicky D., Tomas A., Motsinger-Reif A., Lascelles B.D.X. (2015). Initial evaluation of nighttime restlessness in a naturally occurring canine model of osteoarthritis pain. PeerJ.

[42] Mathias J.L., Cant M.L., Burke A.L.J. (2018). Sleep disturbances and sleep disorders in adults living with chronic pain: A meta-analysis. Sleep Med..

[43] Sakakibara R., Hattori T., Yasuda K., Yamanishi T. (1996). Micturitional disturbance in syringomyelia. J. Neurol. Sci..

[44] St Louis E.K., Jinnur P., McCarter S.J., Duwell E.J., Benarroch E.E., Kantarci K., Pichelmann M.A., Silber M.H., Boeve B.F., Olson E.J. (2014). Chiari 1 Malformation Presenting as Central Sleep Apnea during Pregnancy: A Case Report, Treatment Considerations, and Review of the Literature. Front. Neurol..

[45] Poca M.A., Ferré A., de la Calzada M.D., Moncho D., Fernandez-Torrelles S., Sahuquillo J. (2021). CO2-induced intracranial hypertension and high-amplitude B-waves in a patient with Chiari 1 malformation and sleep apnea syndrome that resolved following CPAP therapy. Acta Neurochir..

[46] Leu R.M. (2015). Sleep-Related Breathing Disorders and the Chiari 1 Malformation. Chest.

[47] Kirjavainen T., Miraftabi P., Martelius L., Karppinen A. (2024). Type one chiari malformation as a cause of central sleep apnea and hypoventilation in children. Sleep Med..

[48] Gagnadoux F., Meslier N., Svab I., Menei P., Racineux J.L. (2006). Sleep-disordered breathing in patients with Chiari malformation: Improvement after surgery. Neurology.

[49] Rabec C., Laurent G., Baudouin N., Merati M., Massin F., Foucher P., Brondel L., Reybet-Degat O. (1998). Central sleep apnoea in Arnold-Chiari malformation: Evidence of pathophysiological heterogeneity. Eur. Respir. J..

[50] Knowler S.P., Dumas E., Spiteri M., McFadyen A.K., Stringer F., Wells K., Rusbridge C. (2020). Facial changes related to brachycephaly in Cavalier King Charles Spaniels with Chiari-like malformation associated pain and secondary syringomyelia. J. Vet. Intern. Med..

[51] Hendricks J.C. (1992). Brachycephalic Airway Syndrome. Vet. Clin. N. Am. Small Anim. Pract..

[52] Pedersen T.R., Bach M.B.T., Stougaard C.L., Gredal H., Rusbridge C., Finnerup N.B., Berendt M. (2024). Clinical predictors of syringomyelia in Cavalier King Charles Spaniels with chiari-like malformation based on owners’ observations. Acta Vet. Scand..

[53] Platt S.R., Olby N.J., British Small Animal Veterinary Association (2013). BSAVA Manual of Canine and Feline Neurology.

[54] Meixensberger J., Baunach S., Amschler J., Dings J., Roosen K. (1997). Influence of body position on tissue-pO2, cerebral perfusion pressure and intracranial pressure in patients with acute brain injury. Neurol. Res..

[55] Gosalakkal J.A. (2008). Sleep-Disordered Breathing in Chiari Malformation Type 1. Pediatr. Neurol..

[56] Olaithe M., Bucks R.S., Hillman D.R., Eastwood P.R. (2018). Cognitive deficits in obstructive sleep apnea: Insights from a meta-review and comparison with deficits observed in COPD, insomnia, and sleep deprivation. Sleep Med. Rev..

[57] Ratcliff R., Dongen H.P.A.V. (2018). The Effects of Sleep Deprivation on Item and Associative Recognition Memory. J. Exp. Psychol. Learn. Mem. Cogn..

[58] Marshall N.S., Wong K.K.H., Cullen S.R.J., Knuiman M.W., Grunstein R.R. (2014). Sleep Apnea and 20-Year Follow-Up for All-Cause Mortality, Stroke, and Cancer Incidence and Mortality in the Busselton Health Study Cohort. J. Clin. Sleep Med..

[59] Xia W., Huang Y., Peng B., Zhang X., Wu Q., Sang Y., Luo Y., Liu X., Chen Q., Tian K. (2018). Relationship between obstructive sleep apnoea syndrome and essential hypertension: A dose–response meta-analysis. Sleep Med..

[60] Lam B., Ryan C.F. (2000). Arnold-Chiari malformation presenting as sleep apnea syndrome. Sleep Med..

[61] Pasterkamp H., Cardoso E.R., Booth F.A. (1989). Obstructive Sleep Apnea Leading to Increased Intracranial Pressure in a Patient with Hydrocephalus and Syringomyelia. Chest.

[62] Sutton B.C., Opp M.R. (2014). Sleep Fragmentation Exacerbates Mechanical Hypersensitivity and Alters Subsequent Sleep-Wake Behavior in a Mouse Model of Musculoskeletal Sensitization. Sleep.

[63] Vanini G., Nemanis K., Baghdoyan H.A., Lydic R. (2014). GABAergic transmission in rat pontine reticular formation regulates the induction phase of anesthesia and modulates hyperalgesia caused by sleep deprivation. Eur. J. Neurosci..

[64] Rusbridge C. (2020). New considerations about Chiari-like malformation, syringomyelia and their management. Practice.

[65] Valentino A., Olby N. (2025). Owner-observed sleep disturbances between Cavalier King Charles Spaniels with and without symptomatic Chiari-like malformation [Abstract N10]. J. Vet. Intern. Med..

