# Supplementary material for: Owner Observed Sleep Disturbances in Cavalier King Charles Spaniels with and Without Clinical Signs Compatible with a Chiari-like Malformation
Source: Animals (Basel). 2026 Jul 14;16(14):2184. doi: 10.3390/ani16142184 (PMC13405612; doi:10.3390/ani16142184)
Supplement: Supplementary file 1 [file animals-16-02184-s001.zip › animals-4369397-supplementary/Supplementary File S1.pdf]

Sleep Study

Thank you for taking the time to take this survey to help better our understanding of sleep disturbances that may exist among dogs with symptoms of Chiari-like malformation/syringomyelia. This should take less than 5 minutes to complete.

You are being asked to complete a survey for research purposes. This survey will ask questions regarding your dogs' medical history and sleeping patterns. You will be volunteering this information to be included in our study. There is no financial compensation for completing this survey and participation is completely voluntary. This survey is confidential and only aggregated data will be reported, meaning your responses will not be identifiable in any reports. If you have any questions regarding this survey please contact ckcschiari@gmail.com. If you agree to continuing with this survey, please select yes.

- ○ Yes, I do consent to completing this survey.
- ○ No, I do not consent to completing this survey

Skip To: End of Survey If Answer = No, I do not consent to completing this survey.

What is your name?

____

What is your dog's name?

____

How old is your dog?

____

Is your dog a Cavalier King Charles Spaniel?

- ○ Yes
- ○ No

What sex is your dog?

- ○ Male Neutered
- ○ Female Spayed
- ○ Male Intact

○ Female Intact

Has your dog been diagnosed with Chiari-like malformation?

○ Yes

○ No

Has your dog been diagnosed with syringomyelia?

○ Yes

○ No

If your dog been diagnosed with Chiari-like malformation? = Yes.
How was your dog diagnosed with Chiari-like malformation/syringomyelia?

○ Diagnosed on MRI

○ Veterinarian suspects based on clinical signs

Does your dog have any other medical conditions? If none, please respond N/A.

____

What medications is your dog currently taking? If none, please respond N/A.

____

Is your dog receiving regular flea/tick preventatives?

○ No

○ Yes

Does your dog have any signs of skin disease?

○ No

○ Yes

The following questions from the CHASE questionnaire will allow us to determine the severity of your dog's signs related to Chiari-like malformation/syringomyelia over the past 7 days.

Scratching

- ○ 0 (not at all)
- ○ 1
- ○ 2
- ○ 3
- ○ 4
- ○ 5
- ○ 6 (could not be more)

Anxious (displaying nervousness or worry)

- ○ 0 (not at all)
- ○ 1
- ○ 2
- ○ 3
- ○ 4
- ○ 5
- ○ 6 (could not be more)

Sensitive (easily upset or hurt)

- ○ 0 (not at all)

- ◯ 1
- ◯ 2
- ◯ 3
- ◯ 4
- ◯ 5
- ◯ 6 (could not be more)

Uncomfortable (displaying signs of physical discomfort)

- ◯ 0 (not at all)
- ◯ 1
- ◯ 2
- ◯ 3
- ◯ 4
- ◯ 5
- ◯ 6 (could not be more)

Restless (unable to relax)

- ◯ 0 (not at all)
- ◯ 1
- ◯ 2
- ◯ 3
- ◯ 4
- ◯ 5

○ 6 (could not be more)

The SNORE 3.0 questionnaire is composed of 6 questions. These will allow us to gain a better understanding on differences in sleeping that may exist among dogs' with Chiari-like malformation.

Over the last 7 days, what has been your dog's ability to sleep at bed time.

○ 1 (falls asleep easily at bedtime)

○ 2

○ 3

○ 4

○ 5

○ 6

○ 7

○ 8

○ 9

○ 10 (takes much longer than usual to fall asleep)

Over the last 7 days, what has been your dog's ability to sleep continuously during the night.

○ 1 (never gets up/paces)

○ 2

○ 3

○ 4

○ 5

- 6
- 7
- 8
- 9
- 10 (constantly getting up/pacing)

Select which option best describes your dog's sleep over the last 7 days.

- 1 (never needs to eliminate during the night)
- 2
- 3
- 4
- 5
- 6
- 7
- 8
- 9
- 10 (needs to eliminate several times every night)

Select which option best describes your dog's breathing while sleeping over the last 7 days.

- 1 (never pauses breathing)
- 2
- 3
- 4

○ 5

○ 6

○ 7

○ 8

○ 9

○ 10 (has several breathing interruptions)

Select the option that best describes how much did vocalizations affect your dog's sleep over the last 7 days.

○ 1 (never wakes him/her up)

○ 2

○ 3

○ 4

○ 5

○ 6

○ 7

○ 8

○ 9

○ 10 (vocalizations are so loud or frequent that constantly wakes him/her up)

Select the option that best describes how much did twitching affect your dog's sleep over the last 7 days.

○ 1 (never wakes him/her up)

○ 2

○ 3

○ 4

○ 5

○ 6

○ 7

○ 8

○ 9

○ 10 (twitches are so frequent and/or severe that constantly wakes him/her up)

Where does your dog sleep at night? Please indicate if they are usually on the bed, in a crate/kennel/bed in the same room as you, or in a separate area of the house.

____

Over the last 7 days how much has your dog snored at night?

○ 1 (never snores)

○ 2

○ 3

○ 4

○ 5

○ 6

○ 7

○ 8

○ 9

◯ 10 (constantly snores)

If your dog has been put on medication (gabapentin, pregabalin, omeprazole) for symptoms associated with Chiari-like malformation, has his/her sleep changed? If your dog has not been diagnosed with Chiari-like malformation, please select not applicable.

◯ Yes

◯ No

◯ Not applicable

If your dog has been put on medication (gabapentin, pregabalin, omeprazole) for symptoms associated... = Yes. Please describe what changes you have seen in your dog's sleep patterns since starting medication.

____

Does your dog tend to sleep with his/her head propped up and elevated?

◯ Yes

◯ No

Are there any other abnormalities you would like us to know about your dog's sleeping patterns? Please describe.

____
