# Supplementary material for: Owner Observed Sleep Disturbances in Cavalier King Charles Spaniels with and Without Clinical Signs Compatible with a Chiari-like Malformation
Source: Animals (Basel). 2026 Jul 14;16(14):2184. doi: 10.3390/ani16142184 (PMC13405612; doi:10.3390/ani16142184)
Supplement: Supplementary file 1 [file animals-16-02184-s001.zip › animals-4369397-supplementary/Supplementary File S2.pdf]

Age	Sex	Has your dog been diagnosed with Chiari-like malformation?	Has your dog been diagnosed with syringomyelia?	Taking Gabapentin or Pregabalin	Taking Pain Medication	Current Medical Conditions	Medications	Scratching	Anxious (displaying nervousness or worry)
2 years	Male Neutered	No	No	No	No	None	None	1	0
7 years	Male Neutered	Yes, MRI	No	No	No	MMVD	Pimobendan, Enalapril, Spironolactone	1	0
9 years	Male Neutered	No	No	No	No	PLE, deaf, murmur	Pred, pimobendan, Atopica	0	2
6 years	Female Spayed	No	No	No	No	KCS	Topical eye medications - several	1	1
1 year	Female Spayed	No	No	No	No	None	None	1	0
2 years	Male Neutered	No	No	No	No	None	None	0	0
10 years	Male Neutered	No	No	No	No	MMVD	None	0	0
3 years	Female Spayed	No	No	No	No	None	Fortiflora, myos, dasuquin, antinol	0	0
3 years	Female Intact	No	No	No	No	None	None	0	0
2 years	Female Spayed	No	No	No	No	None	None	0	0
5 years	Female Spayed	No	No	No	No	EPI, urinary incontinenc e	Incurin, enzymes for EPI, crananidin, coballequin	1	0

1 year	Female Intact	Yes, MRI	Yes	No	No	None	Supplements	0	0
4 years	Female Spayed	Yes, MRI	Yes	Yes	Yes	PSOM	Pregabalin	4	1
3 years	Female Spayed	Yes, MRI	Yes	Yes	Yes	None	Pregabalin	2	0
5 years	Female Spayed	Yes, MRI	Yes	No	Yes	None	Carprofen	4	2
5 years	Female Spayed	Yes, MRI	Yes	Yes	Yes	Dry eyes	Pregabalin, cyclosporine and tacrolimus eye drops	3	2
5 years	Male Neutered	Yes, MRI	Yes	Yes	Yes	None	Pregabalin, Tramadol PRN	3	2
6 years	Female Spayed	Yes, MRI	No	No	No	None	None	1	3
5 years	Female Spayed	Yes, MRI	Yes	Yes	Yes	PSOM; Macrothrombocytopenia	Pregabalin	4	1
3 years	Male Neutered	Yes, MRI	No	Yes	Yes	None	Pregabalin, Tramadol	1	2
4 years	Female Spayed	Yes, MRI	Yes	Yes	Yes	None	Gabapentin, cbd oil pills	4	2

4 years	Female Spayed	Yes, MRI	Yes	Yes	Yes	None	Gabapentin	5	4
3 years	Female Spayed	Yes, MRI	Yes	No	No	None	None	2	3
4 years	Female Spayed	No	No	No	No	None	None	0	0
3 years	Female Spayed	Yes, MRI	No	Yes	Yes	None	Gabapentin	4	4
10 years	Male Neutered	Yes, MRI	Yes	Yes	Yes	MMVD, arthritis	Pimobendan, Rimadyl, Pregabalin, probiotic	0	3
3 years	Female Spayed	Yes, MRI	Yes	Yes	Yes	Loose and soft stool	Pregabalin	3	1
4 years	Male Neutered	Yes, Suspected	Yes	Yes	Yes	None	Gabapentin and Omeprazole	1	1
5 years	Male Neutered	No	No	No	No	None	None	2	3
9 years	Male Neutered	No	No	No	No	Deaf and a heart murmur.	None	3	2
4 years	Female Spayed	No	No	No	No	None	None	1	5
6 years	Male Neutered	No	No	No	No	None	None	5	3
4 years	Male Neutered	No	No	No	No	Fly catchers	Trazodone, Fluoxetine	1	6
5 years	Male Neutered	No	No	No	No	None	None	1	0
5 years	Male Intact	No	No	No	No	None	None	1	0
2 years	Female Spayed	No	No	No	No	None	None	2	0
5 years	Male Neutered	No	No	No	No	None	None	0	0

Sensitive (easily upset or hurt)	Uncomfortable (displaying signs of physical discomfort)	Restless (unable to relax)	CHASE Score	Clinical Signs of CM+/-SM	Falls Asleep At Bedtime	Paces/Continuous Sleep	Eliminates	Pauses Breathing	Vocalizations	Twitching
0	0	0	1	No	1	1	4	1	1	1
0	1	0	2	No	1	1	1	2	1	1
0	0	0	2	No	1	1	1	2	1	1
3	0	0	5	No	1	1	1	2	1	1
0	0	0	1	No	1	2	2	1	1	1
0	0	0	0	No	1	1	1	1	1	1
0	0	1	1	No	1	1	1	1	1	1
0	0	0	0	No	1	1	3	1	1	1
0	0	0	0	No	1	2	1	1	1	1
2	0	0	2	No	1	1	1	1	1	1
0	0	0	1	No	1	2	1	1	1	1

0	0	0	0	No	1	1	2	1	1	1
3	3	2	13	Yes	1	4	4	8	1	1
1	0	0	3	No	1	1	1	1	1	1
2	3	0	11	Yes	1	2	3	1	1	1
2	3	0	10	Yes	1	2	2	1	1	1
5	2	2	14	Yes	1	3	2	6	2	1
4	3	2	13	Yes	1	1	1	2	2	2
3	4	4	16	Yes	8	8	1	2	5	4
2	1	2	8	Yes	2	2	1	2	1	2
4	4	2	16	Yes	3	5	2	7	2	3

3	5	0	17	Yes	1	1	1	1	1	1
3	1	1	10	Yes	2	1	1	1	2	1
0	0	0	0	No	1	1	1	1	1	1
4	3	3	18	Yes	7	2	2	1	2	2
6	3	1	13	Yes	1	1	1	1	1	1
1	2	0	7	Yes	1	2	1	2	1	1
2	1	1	6	No	2	2	1	5	2	3
1	0	1	7	Yes	1	1	1	1	1	1
1	1	2	9	Yes	1	2	1	1	1	1
5	4	2	17	Yes	3	2	9	2	2	2
3	2	1	14	Yes	4	6	6	6	4	4
6	1	0	14	Yes	1	7	2	1	6	1
3	0	0	4	No	1	1	1	1	1	1
2	1	3	7	Yes	4	6	3	3	4	5
3	0	0	5	No	1	3	1	1	1	1
0	0	0	0	No	1	2	1	1	1	1

SNORE Total	Sleep Quality Category	Sleep Interuptions Category	Snoring	Head Propped	Change in sleep with medication	Please describe what changes you have seen in your dog's sleep patterns since starting medication.
9	7	2	8	Yes	Not applicable	
7	5	2	2	No	Not applicable	
7	5	2	6	No	No	
7	5	2	3	Yes	Not applicable	
8	6	2	1	Yes	Not applicable	
6	4	2	1	No	Not applicable	
6	4	2	10		No	
8	6	2	1	No	Not applicable	
7	5	2	3	No	No	
6	4	2	5	No	Not applicable	
7	5	2	1	No	Not applicable	

7	5	2	8	No	Not applicable	
19	17	2	10	No	Yes	She sleeps more soundly, but she wakes earlier than she ever has. Wakes at about 7/7:30 and scratches constantly until we get up.
6	4	2	2	No	No	
9	7	2	5	Yes	Not applicable	
8	6	2	10	No	No	
15	12	3	10	Yes	Yes	He def sleeps better and is not waking up scratching in the middle of the night.
9	5	4	2	No	Not applicable	
28	19	9	9	Yes	Yes	Sleeps more deeply (like she exhausted); wakes more often; wakes more abruptly (like she's scared or hurt).
10	7	3	2	Yes	Yes	More restful sleep; does not need to get up and "resettle" into a different position.
22	17	5	5	No	No	

6	4	2	8	Yes	No	
8	5	3	1	No	No	
6	4	2	1	No	Not applicable	
16	12	4	3	Yes	Yes	More and deeper sleep periods during the day; desire to go to sleep for the night earlier.
6	4	2	3	Yes	Yes	He sleeps much better. Very calm and comfortable since starting the pregablin.
8	6	2	7	Yes	Yes	I think she sleeps better.
15	10	5	8	Yes	Yes	Sleepier.
6	4	2	3	No	Not applicable	
7	5	2	8	No	Not applicable	
20	16	4	2	Yes	No	
30	22	8	10	Yes	No	
18	11	7	5	Yes	Not applicable	
6	4	2	2	No	Not applicable	
25	16	9	2	No	No	
8	6	2	2	No	Not applicable	
7	5	2	3	No	Not applicable	
